# Microbiome dysbiosis inhibits carcinogen-induced murine oral tumorigenesis

**DOI:** 10.7150/jca.75947

**Published:** 2022-08-08

**Authors:** Yuh-Ling Chen, Kuan-Chih Huang, Jer-Horng Wu, Tsunglin Liu, Jiung-Wen Chen, Jia-Yan Xie, Meng-Yen Chen, Li-Wha Wu, Chun-Liang Tung

**Affiliations:** 1 Institute of Oral Medicine, College of Medicine, National Cheng Kung University, Tainan, Taiwan.; 2 Department of Environmental Engineering, National Cheng Kung University, Tainan, Taiwan.; 3 Department of Biotechnology and Bioindustry Sciences, National Cheng Kung University, Tainan, Taiwan.; 4 Institute of Molecular Medicine, College of Medicine, National Cheng Kung University, Tainan, Taiwan.; 5 Department of Oral Maxillo-Facial Surgery, Ditmanson Medical Foundation Chia-Yi Christian Hospital, Chiayi 60080, Taiwan.

**Keywords:** microbiota, oral cancer, antibiotics-induced microbiome dysbiosis, polyamine, spermine oxidase

## Abstract

Oral cancer is one of the most common cancers worldwide and ranks fourth for the mortality rate of cancers in males in Taiwan. The oral microbiota is the microbial community in the oral cavity, which is essential for maintaining oral health, but the relationship between oral tumorigenesis and the oral microbiota remains to be clarified. This study evaluated the effect of microbiome dysbiosis on oral carcinogenesis in mice, and the impact of the microbiome and its metabolic pathways on regulating oral carcinogenesis. We found that antibiotics treatment decreases carcinogen-induced oral epithelial malignant transformation. Microbiome analysis based on 16S rRNA gene sequencing revealed that the species richness of fecal specimens was significantly reduced in antibiotic-treated mice, while that in the salivary specimens was not decreased accordingly. Differences in bacterial composition, including *Lactobacillus animalis* abundance, in the salivary samples of cancer-bearing mice was dramatically decreased. *L. animalis* was the bacterial species that increased the most in the saliva of antibiotic-treated mice, suggesting that *L. animalis* may be negatively associated with oral carcinogenesis. In functional analysis, the microbiome in the saliva of the tumor-bearing group showed greater potential for polyamine biosynthesis. Immunochemical staining proved that spermine oxidase, an effective polyamine oxidase, was upregulated in mouse oral cancer lesions. In conclusion, oral microbiome dysbiosis may alter polyamine metabolic pathways and reduce carcinogen-induced malignant transformation of the oral epithelium.

## Introduction

Oral squamous cell carcinoma (OSCC) is one of the most common human malignancies and ranks fourth in terms of the mortality rate of cancer in males in Taiwan 1. Tobacco, alcohol, and betel nuts are widely considered major risk factors for oral cancer, and emerging studies have also found that poor oral hygiene is associated with a high incidence of oral cancer 2. Despite improvement in surgical techniques and advances in chemotherapy and radiotherapy, the 5-year survival rate of OSCC patients with regional lymph node or other organ metastases remains less than 50%, with no significant improvement over the past 30 years 3,4. Therefore, the prevention of cancer formation, early diagnosis, and development of novel targeted therapies are crucial for oral cancer.

Although cancer is generally considered to be a disease of host genetics and environmental factors, microorganisms are still closely related to human malignancies 5. For example, *Helicobacter pylori* is the main microorganism associated with gastric cancer 6, *Fusobacterium nucleatum* has been indicated as a closely related microbiome to colorectal carcinogenesis 7, and *Streptococcus gallolyticus* has been known to promote colorectal cancer development 8. Although the advances in next-generation sequencing technologies have been used to analyze the microbiome in oral cancer specimens and salivary samples and confirmed that microbial disturbances are associated with OSCC in recent years, no consistent bacterial species or flora has been identified 9-12. Antibiotic-induced microbiome depletion has been used frequently to study the role of the gut microbiome in pathological conditions 13,14. In oral cancer, previous studies have used germ-free mouse models to assess the role of the microbiome in carcinogen-induced oral cancer, but with conflicting results 15,16. Therefore, the effects of antibiotic-induced oral microbiome dysbiosis or depletion on oral carcinogenesis remain to be investigated.

Metabolic pathways or metabolites have been known to be altered in cancer cells and considered critical for the initiation and progression of cancer. The crosstalk of microbial communities and human cells contributes to the maintenance of cellular metabolism. Changes in the balance between the host and microbiota create favorable conditions for cancer development. For example, microbial metabolites can modulate inflammatory responses in the tumor microenvironment or create immunosuppressive conditions that promote cancer development and induce epithelial-mesenchymal transition to promote tumor metastasis 17. Polyamines are small polycationic molecules, which are produced from ingested foods and microbiota metabolism, and are involved in multiple cellular processes, such as gene expression regulation and cell proliferation 18. In cancer, polyamine metabolism is frequently dysregulated by cancer-associated microbes, such as in colorectal cancer 19 and pancreatic cancer 20. However, whether the polyamine metabolism in OSCC is disturbed by microbial alterations needs to be further clarified. In this study, we aimed to investigate the effects of microbiome dysbiosis on oral carcinogenesis in mice and to decipher the impact of the microbiome and its metabolic pathways on regulating oral carcinogenesis.

## Materials and Methods

### Animal experiments and Specimen Collection

**Figure 1:**
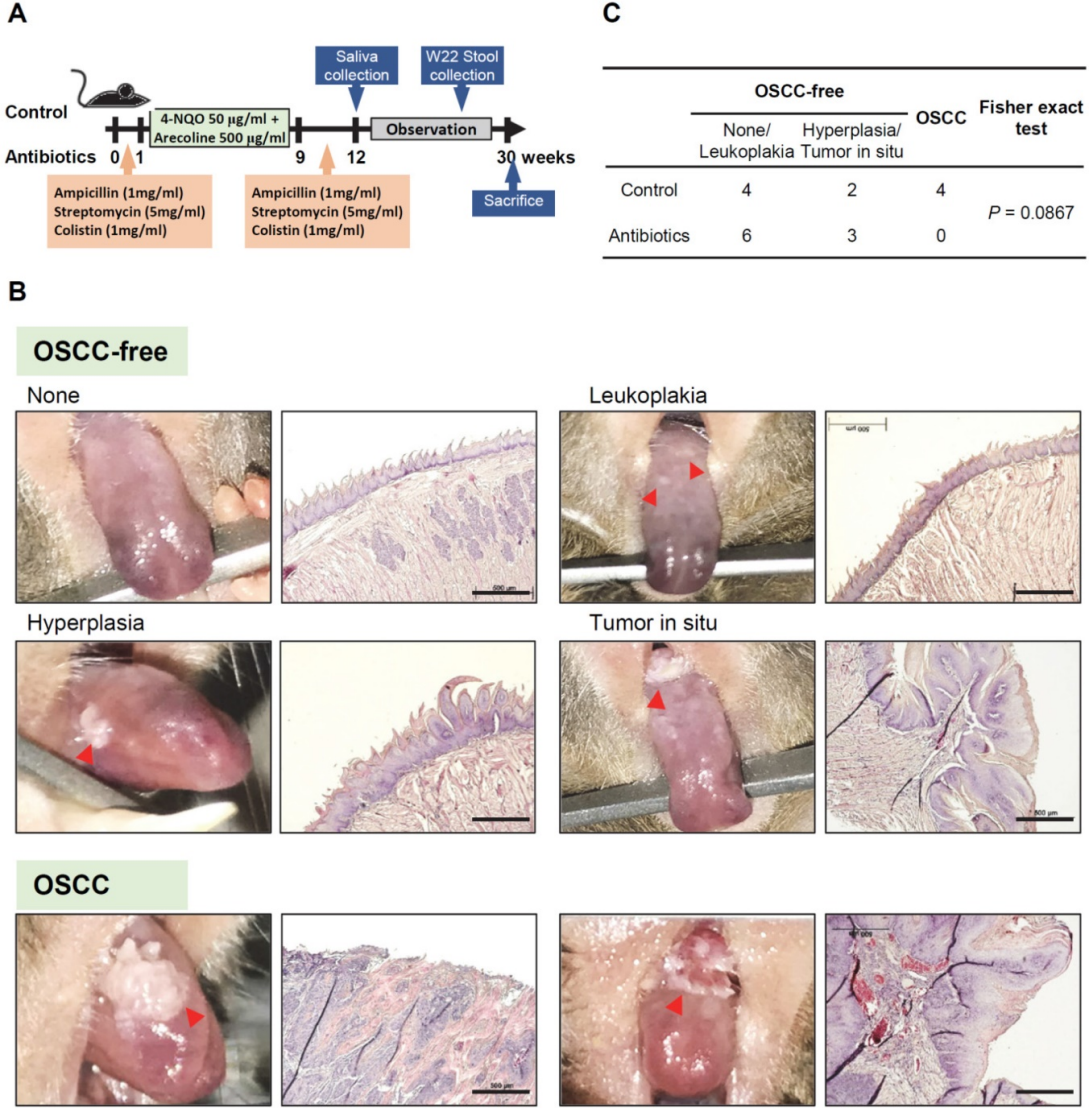
** Antibiotic-induced microbiome dysbiosis decreases 4-NQO/arecoline-induced murine oral tumorigenesis.** (**A**) Schematic diagram of the experimental protocol of antibiotic treatment and 4-NQO/arecoline-induced oral cancer in mice. (**B**) Representative images of mouse tongues without OSCC and with OSCC lesions, and histological staining of tongue tissue sections. Scale bar: 500 µm. (**C**) Incidence of oral lesions in mice. Fisher's exact test was used to analyze the *P* value of the incidence of oral lesions in the two groups.

All animal care and experiments protocols were approved by the Institutional Animal Care and Use Committee (IACUC), NCKU, and complies with ARRIVE (Animal Research: Reporting of *In vivo* Experiments) guidelines. A total of twenty 6-week-old C57BL/6 mice (10 males and 10 females) were randomly divided into a control group and an antibiotics treatment group. A brief summary of the administration of 4-nitroquinoline 1-oxide (4-NQO)/arecoline and antibiotics, as well as the collection of saliva and stool samples, is shown in Figure 1A. The antibiotic regimen consisted of a cocktail of three different antibiotics, ampicillin (1 mg/mL), streptomycin (5 mg/mL), and colistin (1 mg/mL), which were dissolved in sterile drinking water 13. Mice in the antibiotic treatment group drank antibiotic-containing water for 1 week and 3 weeks before and after carcinogen treatment, and the water was changed twice per week. All mice underwent induced oral cancer formation for 8 weeks by drinking 4-NQO (50 μg/mL) and arecoline (500 μg/mL) in water, and the water was changed twice per week 21. The murine oral mucosa and tongue were gently wiped with cotton swabs to absorb the saliva and then soaked in 500 μl PBS buffer for further analysis after the 12^th^ week. Stool samples of mice in the control and the antibiotic treatment groups were collected separately at week 22. After the 12^th^ week, the formation of oral tumors was observed and photographed every three days. Observational data from all mice were included in the analysis, except for one mouse in the antibiotic group that died of injury in week 17. At the 30^th^ week, pathological changes in the tongue were observed in most of the mice, and some tumors significantly affected the feeding of the mice and resulted in weight loss of 25%, which was set as the end of the experiment. All mice were euthanized with CO_2_, and their tongues were collected and fixed in 10% formalin for further histological examination. All experiments in the study were single-blind.

### Bacterial genomic DNA extraction

Saliva specimens were collected and centrifuged 1500 rpm to remove cell debris and then harvested the bacteria by spinning 5000 × g for 10 minutes. Resuspended the bacteria with 500 ul PBS, bacterial genomic DNA was isolated with QIAamp DNA Mini Kit (Qiagen, Germany) in accordance with the manufacturer's protocol. Stool samples were randomly collected from the control and antibiotic group mouse cages and dissolved in 500 ul PBS, respectively. The gDNA of mouse feces was extracted by using DNeasy PowerSoil Kit (Qiagen) according to the manufacturer's instructions. The quantity and quality of isolated gDNA were determined using a NanoDrop 2000 (Thermo Scientific, USA).

### Microbiome analysis

The microbial taxonomy of mouse saliva and fecal samples was analyzed by sequencing the V3-V4 hypervariable region of the 16S rRNA gene. 1μg gDNA was first amplified by polymerase chain reaction (PCR) with bacteria-specific 16S rDNA primers (27F 5'-AGAGTTTGATCCTGGCTCAG-3'; 1492R 5'-GGTTACCTTGTTACGACTT-3'). The V3-V4 hypervariable region of 16S rDNA was amplified using the bacterial-specific forward and reverse primer set using the 16S Metagenomic Sequencing Library Preparation Kit (Illumina, San Diego, CA, USA). Indexed adapters were added to the amplicons using the Nextera XT Index Kit (Illumina, San Diego, CA, USA), according to the manufacturer's instructions. The amplified DNA sizing accuracy was checked using the 4200 TapeStation System (Agilent Technologies, Santa Clara, CA, USA). After library construction, samples were mixed with MiSeq Reagent Kit v3 (600-cycle) and loaded onto a MiSeq cartridge, then a 2x300 bp paired-end sequencing run was performed using the MiSeq platform (Illumina, San Diego, CA, USA). Sample preparation, library construction and sequencing were performed by Welgene Biotech Co., Ltd. (Taipei, Taiwan).

### OTU Analysis & Annotation

The paired-end raw FASTQ reads generated from Illumina MiSeq platform were filtered to remove the Illumina PhiX Control using Bowtie 2 22. Trimmomatic was used to remove sequences with average QV<20 to produce clean reads 23. These clean paired-end reads were merged by overlaping sequences using FLASH 24; then low-quality tails and primers were trimmed and filtered based on length using mothur to produce filtered tags 25. USEARCH was used to remove PCR chimeras to produce effective tags and to construct OTUs (Operational Taxonomic Units) at 97% se-quence identity 26. Taxonomy assignment of OTU sequences was performed using mothur with the SILVA database v132 27. Taxa abundances, rank abundance curves were calculated and plotted using Qiime 28. Rarefaction curves were calculated using the VEGAN R package 29 as were the Beta diversity indices (Bray-Curtis matrix, PCoA and NMDS). OTU analysis and annotation was performed by Welgene Biotech Co., Ltd. (Taipei, Taiwan). Linear discriminant analysis (LDA) was performed using LEfSe (standalone v1.1.2) with default options 30. Before analysis, OTUs annotated as “uncultured” or “unknown” were discarded. When the species could not be determined for an OTU, the species name was replaced by the OTU number. Association between species abundance and OSCC was detected using MaAsLin2 (v1.10.0; options: -t NONE, -f group, -n NONE, -z FALSE, -max_significance 0.25) 31. Differentially abundant species were identified using ANCOM2 (v2.1; options: out_cut=0.05, zero_cut=0.9, lib_cut=1000, p_adjust_method=BH) 32.

### Metabolism Prediction

Open database sources, including the KEGG and MetaCyc pathway database 33, were used to identify metabolic pathways. A heat map of the identified key metabolites was performed by Welgene Biotech Co., Ltd. (Taipei, Taiwan).

### Hematoxylin and Eosin (H&E) staining

Mouse tongue tissues were collected, fixed in 10% formalin, dehydrated and embedded in paraffin. 5 μm-thick tissue sections were sliced and mounted on slides for histological and immunohistochemical (IHC) analysis. Tissue sections were deparaffinized in xylenes 5 minutes twice, 99% ethanol 2 minutes, 95% ethanol 1 minute, 85% ethanol 1minute and 75% ethanol 1 minute in order and washed by ddH_2_O for 1 minute. After ddH_2_O washing, hematoxylin was used to stain the nuclear parts of tissue sections for 15 minutes and washed for 20 minutes. Then, eosin was used to stain the cytoplasm parts of tissue sections for 1 minute and washed for 20 minutes. Images of slides were taken using Olympus BX51 microscope at a magnification of 10X.

### Immunohistochemical staining and assessment

Serial 5-μm histological sections were analyzed using immunohistochemical staining with polyclonal anti-SMOX antibody at the dilution of 1:300 (ab150971, Abcam Inc., MA, USA). Briefly, unstained tissue sections were deparaffinized and rehydrated. By using the heat-induced epitope retrieval method, the tissue sections in retrieval solution (sodium citrate buffer 10 mmol/L, pH 6.0) were heat at 100 °C for 1 hour. Endogenous peroxidase was blocked by 3% hydrogen peroxide solution. Tissue sections were incubated with primary antibody overnight at 4 °C. Secondary antibodies were incubated for 1 hour using a Starr Trek Universal HRP Detection Kit (STUHRP700L10 kit, Biocare Medical, Concord, CA). For the negative controls, the primary antibody was omitted. DAB was used for examining the signal with hematoxyllin counterstaining. Slide examination was carried out using an Olympus BX51 light microscope. Expression of SMOX in the cytoplasm of tumor cells or tongue epithelial cells was recorded. Positive immune-stained cells were counted manually by two independent investigators. The following parameters were used to determine the degree of expression of immunostaining: if less than 10% of cells stained, the specimen was considered negative, while 10%-30%, 31%-70%, and 71%-100% were classified as weak, moderate, and strong expression, respectively.

## Results

### Antibiotic-induced Microbiome Dysbiosis Decreases 4-NQO/arecoline-induced Murine Oral Tumorigenesis

To establish the animal model of oral cancer, 4-NQO and arecoline were added to drinking water to induce tumorigenesis in the oral cavity of mice. Figure 1A shows a schematic diagram of the experimental protocol of antibiotic treatment and 4-NQO/arecoline-induced oral cancer in mice (Figure 1A). C57BL/6 mice were randomly divided into the control and antibiotic-treated groups, with five male and five female mice in each group. After histological examination, we divided the mouse tongue tissue lesions into OSCC-free (including no lesion, leukoplakia, epithelial hyperplasia, and tumor *in situ*) and OSCC groups (Figure 1B). We found that there were six and nine OSCC-free mice in the control and antibiotic groups, respectively, and one of the antibiotic-treated mice did not develop any tumors (Supplementary Figure S1 and Figure 1C). Interestingly, invasive OSCC occurred only in the control mice (*n* = 4) and not in any of the antibiotic group mice (Supplementary Figure S1 and Figure 1C). Although Fisher's exact test analysis showed no statistically significant difference in oral cancer lesions between the control and antibiotic mice (*p* = 0.0867), the incidence of oral cancer lesions appeared to be reduced in the antibiotic-treated mice (Figure 1C).

### Changes in the Microbiome in Mice Feces and Saliva after Antibiotic Treatment

**Figure 2:**
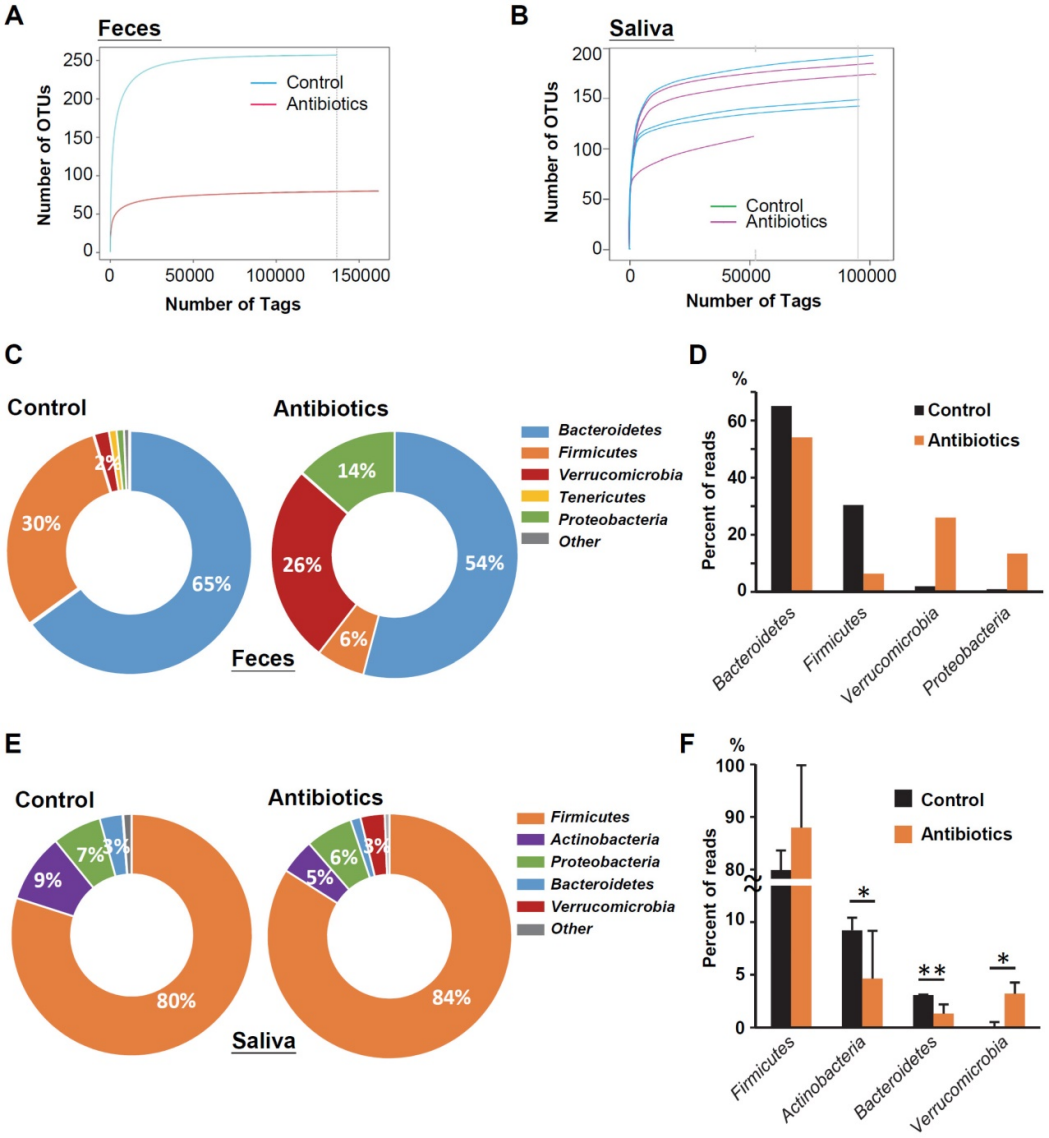
Changes in the microbiome in mice feces and saliva after antibiotic treatment. (**A**) Rarefaction curves of operational taxonomic units (OTUs) diversity for the stool samples. Compared with the control group, the number of OTUs in the antibiotic group was significantly reduced. (**B**) Rarefaction curves of OTUs diversity for the salivary samples. (**C**) The percentage of bacterial phyla in the feces of mice. The top five bacterial phyla are shown. (**D**) The percentage change in the top four bacterial phyla in the feces of mice in the antibiotic group and the control group. (**E**) The percentage of bacterial phyla in the saliva of OSCC-free mice. The top five bacterial phyla are shown. (**F**) The percentage change in the top four bacterial phyla in the saliva of mice in the antibiotic group and the control group.

To determine microbial changes in the antibiotic-treated mice, we analyzed the V3-V4 hypervariable region of the 16S rRNA gene in murine feces and saliva. Rarefaction curves of operational taxonomic units (OTUs) indicated lower species richness and species evenness in the antibiotic-treated murine feces compared with the control mice (Figure 2A). However, the species richness in the murine saliva specimens did not change significantly with antibiotic treatment (Figure 2B). *Bacteroidetes* (65.0%) was the most abundant phylum, followed by *Firmicutes* (30.4%), *Verrucomicrobia* (2.0%), and *Tenericutes* (1.0%) in the feces of control mice (Figure 2C). After antibiotic treatment, the two increased bacterial phyla in the mouse feces were *Verrucomicrons* (2.0% vs. 26.0%) and *Proteobacteria* (1.0% vs. 13.4%), while the decreased bacterial phylum were *Bacteroidetes* (65.0% vs. 54.1%) and *Firmicutes* (30.4% vs. 6.4%) (Figure 2D). *Firmicutes* (79.9%) was the most abundant phylum in the saliva of the control mice, followed by *Actinobacteria* (9.2%), *Proteobacteria* (6.6%), and *Bacteroidetes* (3.1%) (Figure 2E). Intriguingly, similar to the mouse feces specimens, anti-biotic treatment also increased the abundance of *Verrucomicrons* (0.1% vs. 3.2%) in mouse saliva, but the increase in *Firmicutes* abundance (79.9% vs. 84.0%) was opposite to the change in mouse feces (Figure 2F).

### Characterization of the Microbiome in the Saliva of Oral Tumor-bearing Mice

**Figure 3:**
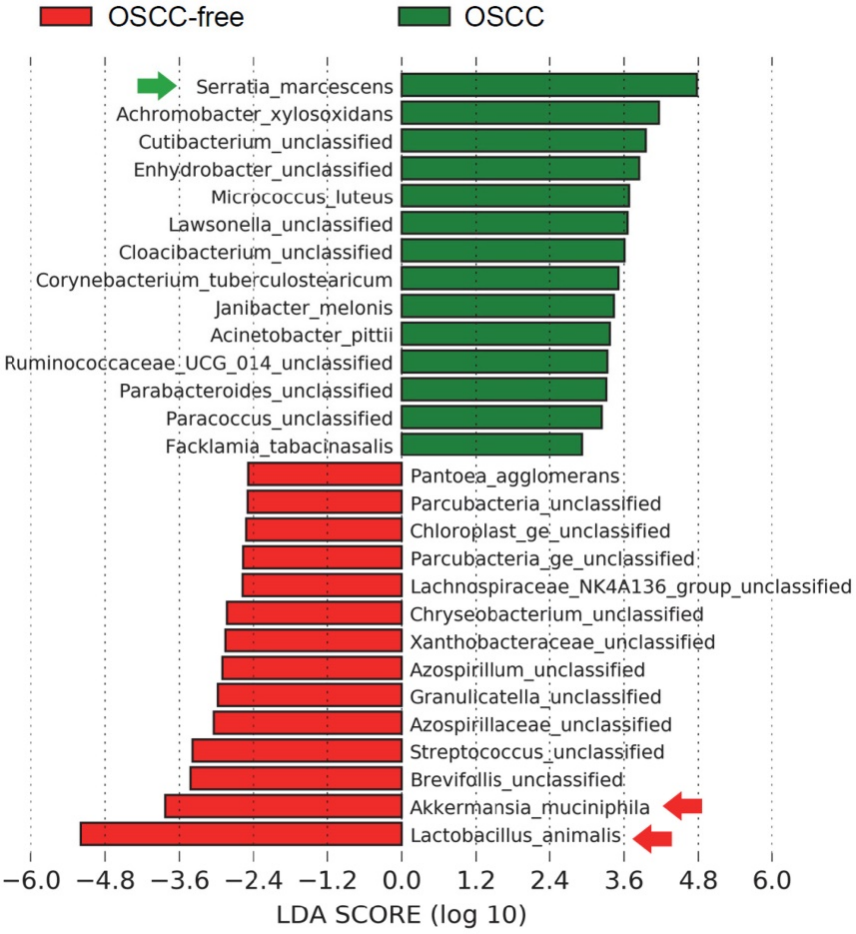
Characterization of microbiomes in the saliva of OSCC-bearing mice. Linear discriminant anal-ysis (LDA) effect size (LEfSe) analysis performed on the microbial community relative abundance data in the saliva microbiota of the control group's OSCC versus OSCC-free mice.

We further sought to determine the taxa in murine saliva that contribute to tumor formation; intergroup differences at the species level in each group were analyzed by the linear discriminant analysis (LDA) effect size (LEfSe) method 30. The salivary microbial communities in mice with oral tumors were first compared to those in mice without oral tumors. A histogram of the LDA scores showed differential abundance in the saliva between tumor-bearing mice and tumor-free mice. LEfSe revealed 23 species that might ex-plain differences between the tumor-bearing and tumor-free mice (Figure 3). Among those, *Lactobacillus animalis* was the most depleted species in the tumor-bearing group. This species was also found associated with OSCC using MaAsLin2 (Supplementary Figure S2 and Table S1). Interestingly, we found that the same a bacterial species *L. animalis* was also more enriched in the antibiotic treatment group (Supplementary Figure S3), indicating that antibiotics may reduce the incidence of oral cancer due to the increase in *L. animalis*.

### Changes in the Metabolic Pathways of the Oral Tumor-associated Microbiome

**Figure 4:**
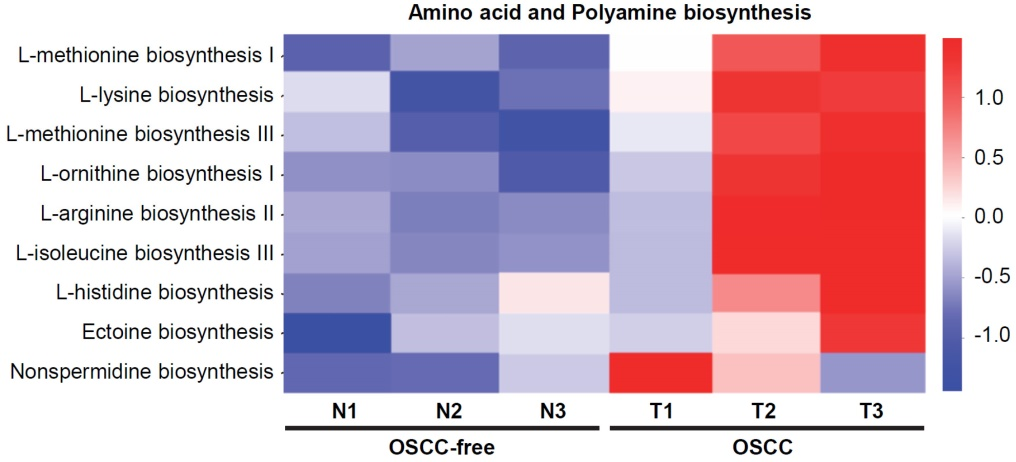
** Predicted functional composition of metagenomes based on 16S rRNA gene sequencing data of murine salivary samples.** Heat map of differentially abundant Kyoto Encyclopedia of Genes and Genomes (KEGG) pathways identified in the six specimens. The values of color in the heat map represent the normalized relative abundance of KEGG pathways.

Bacterial metabolites are important regulators and may exert important influences on the biological functions of host cells. Here, we further analyzed the changes in the metabolic pathways of the microbiome in the saliva of tumor-bearing mice. Kyoto Encyclopedia of Genes and Genomes database (KEGG) pathway enrichment analysis of differentially microbial communities from OSCC-bearing mice (T1-T3) vs. OSCC-free mice (N1-N3) was performed. The results showed that the amino acid (such as methionine, lysine, iso-leucine, and histidine) and polyamine (such as arginine, ornithine, and nonspermidine) biosynthesis pathways were most enriched in OSCC-bearing mice (Figure 4).

### Oral Tumor-associated Microbiome Altered Polyamine Biosynthesis May Promote the Expression of Spermine Oxidase (SMOX) in Murine Oral Cancerous Tissues

**Figure 5:**
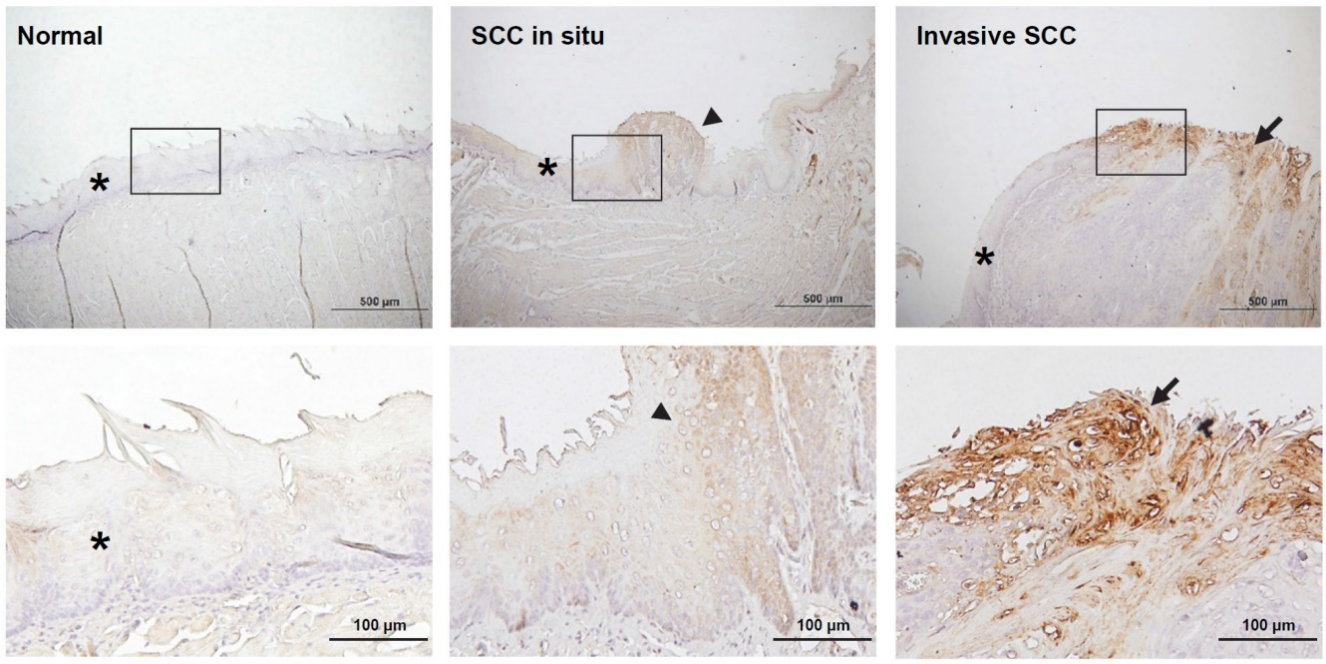
** Immunohistochemical staining of spermine oxidase (SMOX) in mouse normal tongue and tongue cancer tissues.** The rectangular images on the upper panel are enlarged and displayed in the lower panel. The stars, arrowheads, and arrows indicate normal, precancer, and cancer tissues, respectively. Scale bars in upper panel = 500 µm, scale bars in lower panel = 100 µm.

The analysis of metabolic pathways in the salivary microbiome showed that the biosynthesis of polyamines was increased in the saliva microbiome of mice with oral cancer, so we were interested in whether the expression of polyamine metabolism-related enzymes in mouse oral cancer tissues is also changed. Spermine oxidase (SMOX) is a polyamine metabolizing enzyme that catalyzes the conversion of spermine to spermidine and is known to mediate *Helicobacter pylori*-induced gastric cancer 34. Here, we carried out immunohistochemistry (IHC) for SMOX on murine oral mucosal tissues. We found that SMOX immunostaining increased with the progression of oral cancer; it was barely expressed in normal tongue epidermal tissues, but showed moderate staining in precancerous lesions and strong staining in cancerous tissues (Figure 5). We also found that the SMOX IHC was more densely stained in the marginal areas of the tongue cancer tissues, suggesting that cells located on the tumor surface may be exposed to external bacteria to induce SMOX expression. We also found that SMOX IHC staining in leukoplakia tissues from antibiotic-treated mice was weaker than in tissues from control mice (Supplementary Figure S4).

## Discussion

Increasing our understanding of the role of the microbiota in carcinogenesis will give new perspectives for future cancer treatment and prevention strategies. In this study, we used a mouse model of oral cancer induced by 4-NQO/arecoline to provide evidence that the dysbiosis of the microbiota affects the incidence of oral cancer. This is the first report on the impact of antibiotics-induced dysbiosis or depletion of the oral microbiome on oral carcinogenesis and finds a cancer-promoting role of the oral microbiome. We determined the characteristics of the oral microbiota and its associated metabolic pathways of tumor-bearing mice and proved that tumor-related microorganisms might upregulate the expression of polyamine metabolizing enzyme SMOX in the host cells.

In this study, we used a mixture of streptomycin, colistin, and ampicillin as a broad-spectrum antibiotic regimen that targeted both Gram-positive and Gram-negative bacteria, in order to clarify the microbiota of oral carcinogenesis. Broad-spectrum antibiotic treatment has been used for the purpose of very significantly ablating the gut microbiota 13. Most animal studies using antibiotics to deplete the microbiota have illustrated the role of the microbiome in promoting different types of cancer. For instance, Ma et al. found that depleting gut commensal bacteria enhanced the primary bile acids in the liver, causing natural killer T cells to accumulate and thereby preventing liver tumorigenesis 35. Antibiotic-mediated gut microbiome depletion also significantly reduced the tumor burden in several genetically engineered and xenograft mouse models, such as pancreatic cancer, colon cancer, lung cancer, and melanoma 36,37. On the other hand, microbial antitumoral effects have also been reported 38. Bacterial toxins and metabolites can prevent tumor growth and activate the immune system, leading to reduced tumor development 38. In oral cancer, using germ-free animal models to evaluate the role of the microbiome in 4-NQO-induced oral cancer, that it did not differ in tumor incidence, diversity, and size, the authors also suggested that the dose of the carcinogen may affect the ability to draw conclusions 15. Stashenko et al. recently reported that directly transplanting the oral microbiome of donor mice (whether normal mice or oral cancer patients) into germ-free mice can promote 4-NQO-induced oral tumors 16. This observation is consistent with our results and proves that oral microbes can promote the formation of oral cancer. Moreover, we found that antibiotic treatment has no significant effect on the richness of oral microbes, but oral tumors seem to reduce the richness of oral microbes. A decrease in the richness and diversity of microbes has been observed in the cancer tissues and saliva of clinical human oral cancer patients 39, and it has also been found in the saliva specimens of patients with oral precancerous lesions 40.

The present study demonstrated, for the first time, that *L. animalis* was more enriched in the saliva of tumor-free mice and the antibiotic treatment groups, suggesting that this bacterial species may be associated with the prevention of oral cancer development. The anti-cancer activity of *Lactobacillus* strains has received considerable attention as a beneficial microbiota. For example, culture supernatants of *L. acidophilus* exhibited a significant inhibitory effect on the proliferation of human breast cancer cells and tumor growth in xenograft mouse models 41. The treatment with *L. bulgaricus* decreased the tumor volumes in colitis-associated colon cancer and attenuated intestinal inflammation by suppressing the production of IL-6, TNF-α, IL-17, IL-23, and IL-1β 42. *Lactobacillus*-driven down-regulation of the Wnt/β-catenin pathway-related genes was accompanied by tumor growth inhibition, which also suggests that this probiotic can be used as a clinical supplement for the prevention and treatment of colon cancers 43. In the oral cavity, *Lactobacillus* also acts against oral pathogens associated with periodontitis and caries and even induces apoptosis in oral cancer cells 44,45. Although we have identified potential oral microorganisms associated with oral tumorigenesis in mice tumor models, the impact of these bacterial strains on the formation of human oral cancers needs to be further elucidated in the future.

Bacterial metabolites are important regulators of host cell function and may play a critical role in the physiological and pathological conditions of the host. In the present study, we found that the biosynthesis of polyamines was increased in the saliva microbiome of mice with oral cancer. Moreover, the expression of the polyamine metabolizing enzyme SMOX increased with the degree of oral tissue malignant transformation, and there are also more expressions on the surface of oral mucosal tissues that may be frequently exposed to foreign bacteria. A recent study through the integration of metabolomics analysis and transcriptomic data also found that the polyamine pathway is significantly dysregulated in human OSCC, including the upregulation of SMOX 46. Although our data suggested that the increase in SMOX in oral cancer cells may be due to frequent exposure to bacteria with polyamine metabolism, the effect of microorganisms in the up-regulation of SMOX needs to be further investigated. The polyamines are produced through a continuous process that starts with the conversion of L-ornithine to putrescine, and then putrescine is metabolized to spermidine and spermine. SMOX specifically back-converts spermine to spermidine, generating H_2_O_2_ in the process, which leads to DNA damage and subsequent tumorigenesis 18. The induction of SMOX by *Helicobacter pylori* infection mediates *H. pylori*-induced gastric inflammation, DNA damage, and activation of β-catenin signaling in gastric cancer 34. Microbiota and their metabolites participate in the carcinogenic process through different pathological mechanisms, including induction of epithelial cell gene mutations and regulation of the tumor microenvironment 47,48. Further investigating the direct and indirect mechanisms by which the oral microbiota can influence the metabolism in oral keratinocytes as well as the interplay between the host and microbiota metabolic pathways will help with the development of oral cancer treatment and prevention by regulating the microbiota.

## Supplementary Material

Supplementary figures and table.
