# Supplementary material for: Microbiome dysbiosis inhibits carcinogen-induced murine oral tumorigenesis
Source: J Cancer. 2022 Aug 8;13(10):3051–60. doi: 10.7150/jca.75947 (PMC9414028; doi:10.7150/jca.75947)
Supplement: Supplementary file 1 — Supplementary figures and table. [file jcav13p3051s1.pdf]

Supplementary Figure S1. Representative images of oral lesions in mice and histology staining of tongue tissue sections. Scale bar: 500 μm.

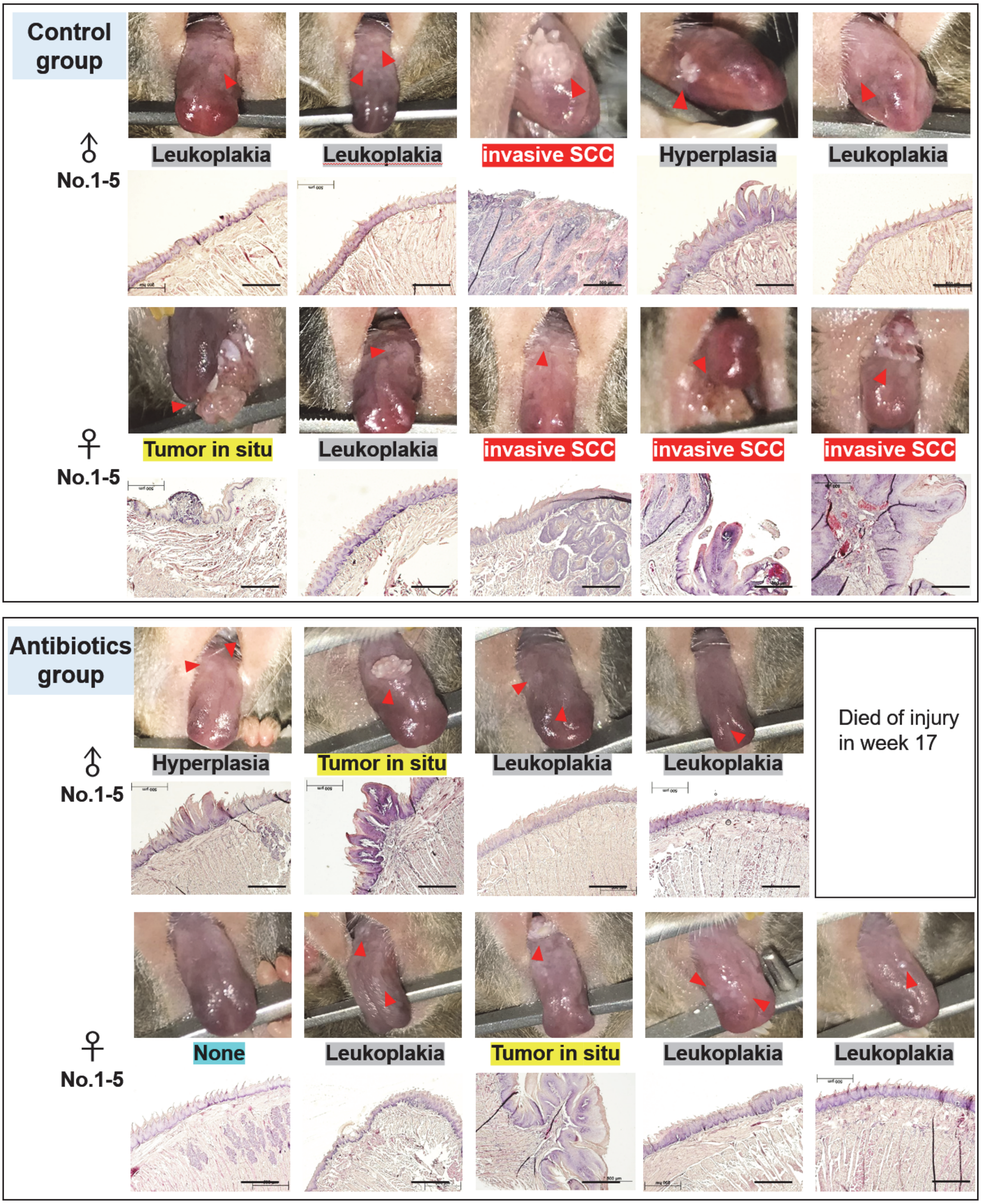

Supplementary Figure S2. Relative abundance of *Lactobacillus animalis* in OSCC and OSCC-free mice using a MaAsLin2 analysis.

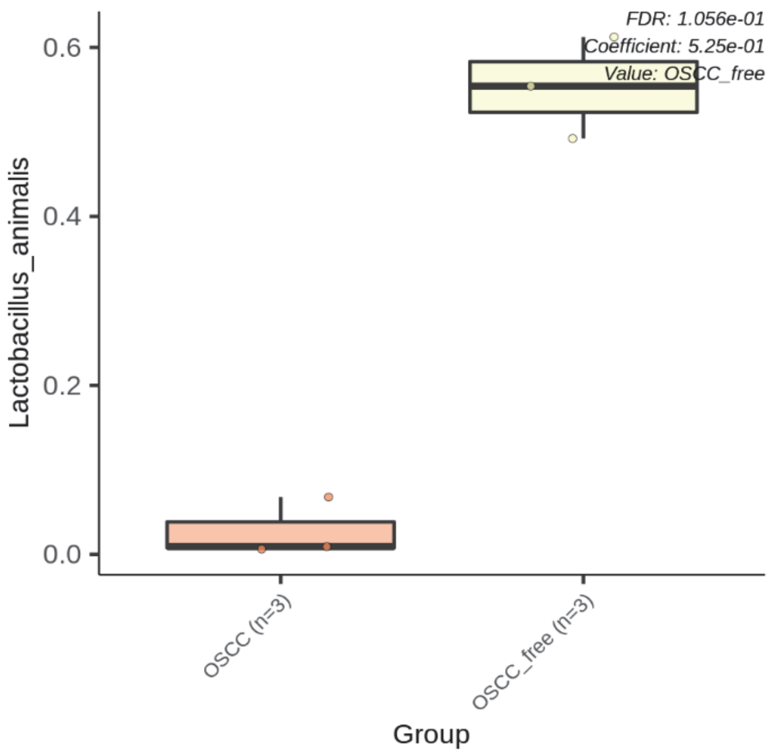

Supplementary Figure S3. Characterization of microbiomes in the saliva of control and antibiotics-treated mice. Linear discriminant analysis (LDA) effect size (LEfSe) analysis performed on the microbial community relative abundance data in saliva microbiota of control versus antibiotic-treated mice. A red arrow indicate bacterial species that was more abundant in both the OSCC-free and antibiotic-treated samples.

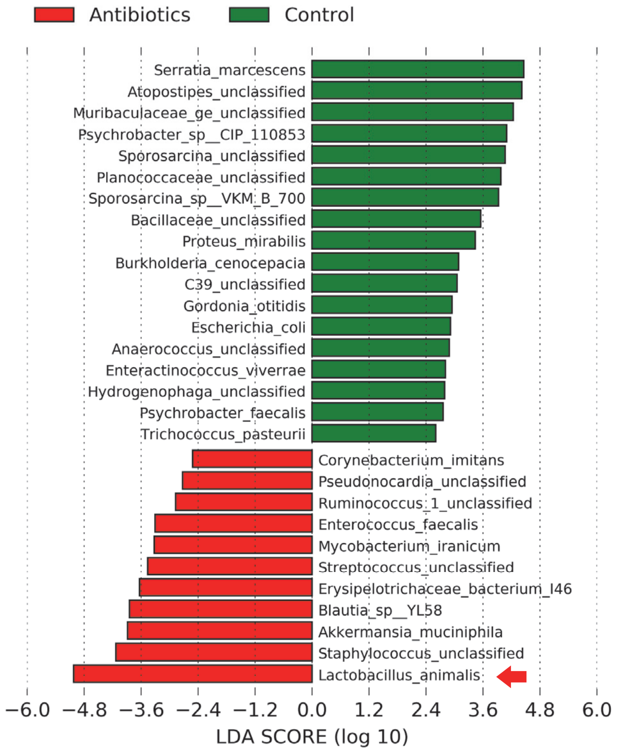

Supplementary Figure S4. Immunohistochemical staining of spermine oxidase (SMOX) in mouse tongue leukoplakia. The rectangular images of the upper panel are enlarged and displayed in the lower panel. Scale bars in upper panel = 500 μm, scale bars in lower panel = 100 μm.

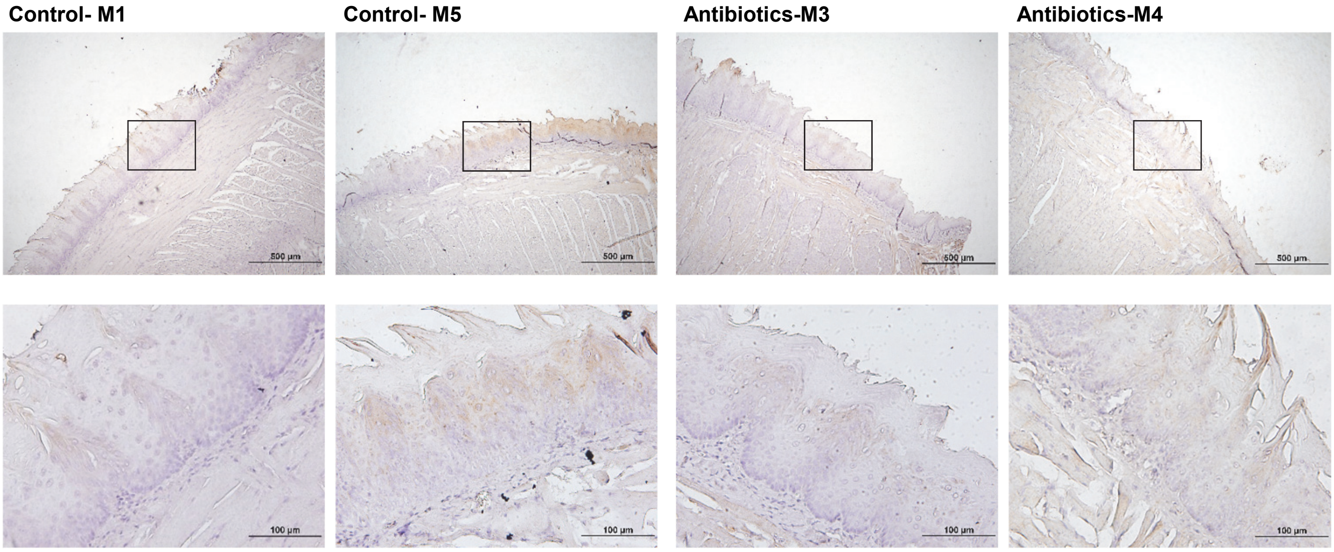

Supplementary Table S1. Bacterial species associated with OSCC identified by MaAsLin2 with a *p*-value <0.2.

Feature	coef.	p-value	q-value
Lactobacillus_animalis	0.52521	0.00020	0.10560
Muribaculaceae_ge_Otu10	-0.02693	0.00393	0.49392
Muribaculaceae_ge_Otu8	-0.02343	0.01360	0.49392
Sphingobium_Otu43	0.00402	0.02148	0.49392
Muribaculaceae_ge_Otu32	-0.01182	0.02387	0.49392
Pseudomonas_Otu55	0.00224	0.03622	0.49392
Brevibacterium_casei	0.00118	0.06427	0.49392
Muribaculaceae_ge_Otu16	-0.00695	0.07832	0.49392
Muribaculaceae_ge_Otu15	-0.01217	0.09301	0.49392
Bacillus_Otu58	-0.00338	0.09329	0.49392
Chloroplast_ge_Otu217	-0.00019	0.09909	0.49392
Sporosarcina_sp._VKM_B.700	-0.01585	0.10957	0.49392
Bacillaceae_Otu47	-0.00556	0.11078	0.49392
Facklamia_tabacinasalis	-0.00235	0.11152	0.49392
Parabacteroides_Otu13	-0.00962	0.11249	0.49392
Fusobacterium_varium	-0.00021	0.11617	0.49392
Pseudomonas_Otu87	0.00093	0.11618	0.49392
Leptotrichia_shahii	-0.00013	0.11619	0.49392
Acidibacter_Otu243	0.00039	0.11653	0.49392
Corynebacterium_imitans	0.00039	0.12242	0.49392
Proteus_mirabilis	-0.00981	0.12343	0.49392
Kocuria_rhizophila	0.00240	0.12922	0.49392
Sphingomonadaceae_Otu149	0.00038	0.13237	0.49392
Lachnospiraceae_Otu567	-0.00026	0.13240	0.49392
Methylobacterium_adhaesivum	-0.00009	0.13399	0.49392
Ruminiclostridium_5_Otu245	-0.00010	0.13758	0.49392
Enteractinococcus_viverrae	-0.00281	0.14006	0.49392
Chryseobacterium_Otu90	0.00068	0.14069	0.49392
Bosea_vestrisii	0.00038	0.14481	0.49392
Cutibacterium_Otu481	-0.00003	0.14703	0.49392
Alistipes_Otu216	-0.00009	0.14705	0.49392
Psychrobacter_sp._CIP_110853	-0.02707	0.14867	0.49392
Ruminococcus_1_Otu76	0.00094	0.14996	0.49392
Acinetobacter_Otu70	0.00719	0.15595	0.49392
Carnobacteriaceae_Otu221	-0.00022	0.16470	0.49392
Paenalcaligenes_Otu41	-0.00229	0.16786	0.49392
Chloroplast_ge_Otu113	0.00035	0.16958	0.49392
Gemella_haemolysans_ATCC_10379	0.00156	0.17110	0.49392
Brevundimonas_nasdae	0.00390	0.17866	0.49392
Pseudogracilibacillus_Otu435	-0.00004	0.18030	0.49392
Alistipes_shahii	-0.00009	0.18083	0.49392
Methylobacterium_Otu444	-0.00004	0.19659	0.49392
Haemophilus_haemolyticus	0.00052	0.19694	0.49392
Escherichia_coli	-0.19137	0.19883	0.49392
